# Discrete phase space-continuous time relativistic Klein–Gordon and Dirac equations, and a new non-singular Yukawa potential

**DOI:** 10.1038/s41598-023-47344-w

**Published:** 2023-11-21

**Authors:** Anadijiban Das, Rupak Chatterjee

**Affiliations:** 1 Department of Mathematics, Simon Fraser University, Burnaby, BC V5A 1S6 Canada; 2 Department of Applied Physics, New York University, 2 MetroTech Center, Brooklyn, NY 11201 USA

**Keywords:** Quantum mechanics, Theoretical particle physics

## Abstract

This paper deals with the second quantization of interacting *relativistic Fermionic and Bosonic fields* in the arena of discrete phase space and continuous time. The mathematical formulation involves *partial difference equations*. The corresponding Feynman diagrams and a new $S^{\#}$-matrix theory is developed. In the special case of proton-proton Møller scattering via an exchange of a neutral meson, the explicit second order element $\langle f | S^{\#}_{(2)} |i \rangle$ is deduced. In the approximation of very low external three-momenta, *a new Yukawa potential* is explicitly derived from $\langle f | S^{\#}_{(2)} |i \rangle$. Moreover, it is rigorously proved that this new Yukawa potential is *divergence-free.* The mass parameter of the exchanged meson may be set to zero to obtain a type of scalar Boson exchange between hypothetical Fermions. This provides a limiting case of a new Coulomb type potential directly from the new singularity free Yukawa potential. A divergence-free Coulomb potential between two Fermions at two discrete points is shown to be proportional to the Euler beta function. Within this relativistic discrete phase space continuous time, a single quanta is shown to occupy the hyper-tori $S^{1}_{n^1} \times S^{1}_{n^3} \times S^{1}_{n^3}$ where $S^{1}_{n}$ is a circle of radius $\sqrt{2n+1}$.

## Introduction

Quantum mechanics has been exactly represented by the standard partial differential equations of Schrödinger, the matrix mechanics of Heisenberg, and the phase space continuous time continuum of Weyl and Wigner^1,2^. Many of their original papers and other formulations of quantum mechanics in phase space may be found in^3,4^. In recent years, an exact representation of quantum mechanics has been introduced in the discrete phase space and continuous time arena^5–9^ with the use of partial difference-differential equations.

The second quantization of relativistic interacting fields in a background space-time continuum has given rise to the usual *S*-matrix theory^10–12^ and its well known problem of divergences and subsequent renormalization attempts. On the other hand, the analogous $S^{\#}$-matrix in the arena of discrete phase space and continuous time has been shown to soften the the degree of divergences^7–9,13^. In fact, in^13^, it was shown the the $S^{\#}$-matrix formulation for a second order electron-electron scattering (or Møller scattering) led, in the low momenta approximation of two external electrons, to a new Coulomb potential that was completely divergence-free.

In this paper, we investigate the problem of a new Yukawa style potential arising from the discrete phase space-continuous time representation of relativistic quantum field theory and the corresponding $S^{\#}$-matrix. We show this new Yukawa style potential is completely divergence-free. In “section Notations and preliminary definitions”, we summarize briefly the notations used in the present paper. “Section Partial difference and differential operators” defines various partial difference operators^7–9,14^ and the corresponding basis of Hermite polynomials^15^. The following three sections describe the second quantization of free relativistic scalar and Fermionic fields and thereafter, interacting fields in the discrete phase space-continuous time $S^{\#}$-matrix formulation^7–13^. The specific example of Møller scattering is investigated.

“Section Discrete phase space and a new non-singular Yukawa potential” describes the new discrete phase space Yukawa-style potential. The Green’s function of the corresponding partial difference equation in such a static scalar field with Yukawa mass parameter $\mu$ is shown to have a zero discrete phase space distance limit of $\mu \; \exp (\mu ^2)\; \Gamma (-1/2, \mu ^2)$ where $\Gamma (-1/2, \mu ^2)$ is a non-singular incomplete gamma function^15^ for $\mu >0$. This divergence free quality is the main attraction of our new Yukawa potential.

In “section Discrete phase space, a new non-singular Coulomb potential, and Beta functions”, we take the $\mu =0$ limit of our new Yukawa potential to find a new non-singular Coulomb potential. The Green’s function of the corresponding partial difference equation is shown to be proportional to the Euler beta function similar to that found in string theory^16^. The coincidence discrete space limit is shown to produce a non-singular result similar to our previous work^13^. We point out that a single field quanta in this relativistic discrete phase space formulation occupy hyper-tori $S^{1}_{n^1} \times S^{1}_{n^3} \times S^{1}_{n^3}$ where $S^{1}_{n}$ is a circle of radius $\sqrt{2n+1}$ as discussed extensively in^17,18^. Finally, “section Various discrete phase space Green’s functions and a new non-singular Yukawa potential” summarizes some of the key results of the paper while illustrating the divergence free nature of our new Yukawa style potential.

Some earlier work on quantum field theory in both discrete space and time variables are by Yamamoto^19^ where difference equations are used for the fundamental field equations. Yamamoto’s equations are different than ours as we have used continuous time. Yamamoto also obtains divergent free results such as ours for strictly massive bosons. Another early attempt in quantum mechanics along the works of Weyl and Mackey is^20^. Here, the authors construct a discrete time evolution operator for their finite space-time lattice for free Hamiltonians. Very recent work using quantum cellular automatons is^21^ where a $(1+1)$ dimensional QED model is constructed using a circuit of unitary gates. Other formulations using both discrete space and time are found in lattice gauge field simulations where Euclidean space (Wick rotated time) is discretized into a lattice with sites separated by some characteristic distance and connected by links where the gauge fields themselves are defined^22^. Monte Carlo simulations are used to solve such models making them different from the theory presented in this paper. To our knowledge, one of the earliest examples of simple cellular models of space-time which incorporated a fundamental length in a natural way using partial difference equations was by the first author of this paper in 1960^23^.

## Notations and preliminary definitions

There exists a characteristic length $l >0$ in this theory, which is conjectured to be the Planck length. We employ fundamental units characterized by $\hbar = c= l =1$. Thus, all mathematical expressions involving physical phenomena appear as physically dimensionless numbers. Greek indices take values from $\{1,2,3,4\}$ whereas the Roman indices take values from $\{1,2,3\}$. Einstein’s summation convention is adopted in both cases. We denote the flat space-time metric by $\eta _{\mu \nu }$ with the corresponding diagonal matrix $[\eta _{\mu \nu }]:= diag[1,1,1,-1]$. Therefore, we use a signature of +2 in this paper. An element of our discrete space phase and continuous time is expressed as $({\textbf{n}},x^4) \equiv (n^1, n^2, n^3, t) \in {\mathbb {N}}^3 \times {\mathbb {R}}, n^j \in {\mathbb {N}}$ for $j \in {1,2,3}$ and $x^4 \equiv t \in {\mathbb {R}}$.

Let a real or complex-valued function *f* from ${\mathbb {N}}^3 \times {\mathbb {R}}$ into ${\mathbb {R}}$ or ${\mathbb {C}}$ be denoted as $f({\textbf{n}}, t) = f(n^1, n^2, n^3, t)$. We denote the first quantized wave function $\phi ({\textbf{n}},t)$ and the second quantized wave function by the same symbol. The context should indicate the quantization order.

## Partial difference and differential operators

We denote various *partial difference operators and the partial differential operators* as follows^7–9,13^

1 $$\begin{aligned} \begin{array}{l} \Delta _j f({\textbf{n}}, t):= f(\ldots ,n^j+1,\ldots , t) - f(\ldots ,n^j,\ldots , t) \\ \\ \Delta _j^{'} f({\textbf{n}}, t):= f(\ldots ,n^j,\ldots , t) - f(\ldots ,n^j-1,\ldots , t) \\ \\ \Delta _j^{\#} f({\textbf{n}}, t):= \dfrac{1}{\sqrt{2}} \left[ \sqrt{n^j+1} f(\ldots ,n^j+1,\ldots , t) - \sqrt{n^j} f(\ldots ,n^j-1,\ldots , t) \right] \\ \\ \Delta _j^{o} f({\textbf{n}}, t):= \dfrac{1}{\sqrt{2}} \left[ \sqrt{n^j+1} f(\ldots ,n^j+1,\ldots , t) + \sqrt{n^j} f(\ldots ,n^j-1,\ldots , t) \right] \\ \\ \partial _t f({\textbf{n}}, t):= \dfrac{\partial }{\partial t} [f({\textbf{n}}, t)] \end{array} \end{aligned}$$

We now introduce Hermite polynomials and some pertinent properties by the following equations^7–9,15^,

2 $$\begin{aligned} \begin{array}{l} H_{n^j}(k_j):= (-1)^{n^j} e^{(k_j)^2} \dfrac{d^{n^j}}{(dk_j)^{n^j}}[e^{-(k_j)^2}] \\ \\ \dfrac{d^2}{(dk_j)^2}[H_{n^j}(k_j)] -2k_j\dfrac{d}{dk_j}[H_{n^j}(k_j)]+2n^j [H_{n^j}(k_j)]=0 \\ \\ \dfrac{d}{dk_j}[H_{n^j}(k_j)] = 2n^j [H_{n^j-1}(k_j)], \; n^j \ge 1 \\ \\ H_{n^j+1}(k_j)=2k_j[H_{n^j}(k_j)]-2n^j[H_{n^j-1}(k_j)], \;\;n^j \ge 1 \end{array} \end{aligned}$$

and the scaled Hermite function

3 $$\begin{aligned} \begin{array}{l} \xi _{n^j}(k_j):= \dfrac{(i)^{n^j} e^{-(k_j)^2/2} H_{n^j}(k_j)}{(\pi )^{1/4}2^{(n^j/2)}\sqrt{(n^j)!}} \\ \\ \displaystyle \int \limits _{{{\mathbb {R}}}^3} \left\{ \prod _{j=1}^3 \left[ \xi _{n^j}(k_j) \overline{\xi _{{\hat{n}}^j}(k_j)}\right] \right\} dk_1 dk_2 dk_3 = \delta _{n^1 {\hat{n}}^1} \delta _{n^2 {\hat{n}}^2} \delta _{n^3 {\hat{n}}^3} =: \delta ^3_{{\textbf{n}} \hat{{\textbf{n}}}} \\ \\ -i \Delta ^{\#} \xi _{n^j}(k_j) = k_j \xi _{n^j}(k_j) \\ \\ -i \Delta ^{\#} \overline{\xi _{n^j}(k_j)} = -k_j \overline{\xi _{n^j}(k_j)} \end{array} \end{aligned}$$

## The second quantization of the free relativistic partial difference-differential scalar wave field equation

The second quantized Klein–Gordon scalar wave field over the discrete phase space-continuous time arena will be denoted as $\phi ({\textbf{n}},t)=\phi ^{\dagger }({\textbf{n}},t)$. It is Hermitian linear operator acting on a Hilbert space bundle. The linear operator $\phi ({\textbf{n}},t))$ satisfies the *partial difference-differential equations*^7–9^:

4 $$\begin{aligned} \delta ^{ab} \Delta _a^{\#}\Delta _b^{ \#}\phi ({\textbf{n}},t) -(\partial _t)^2 \phi ({\textbf{n}},t) -\mu ^2 \phi ({\textbf{n}},t) = 0 \end{aligned}$$

where $\mu >0$ denotes the mass of a neutral scalar Boson. A class of exact plane wave solutions of the above equation is furnished by

5 $$\begin{aligned} \begin{array}{l} \phi ^{(-)}({\textbf{n}},t) = \displaystyle \int \limits _{{{\mathbb {R}}}^3} d^3{\textbf{k}}\,\,\,[2\omega ({\textbf{k}})]^{-1/2} \left\{ a({\textbf{k}}) \left[ \displaystyle {\prod _{j=1}^{3}} \xi _{n^j}(k_j) \right] e^{-i\omega t} \right\} \\ \\ \phi ^{(+)}({\textbf{n}},t) = \displaystyle \int \limits _{{{\mathbb {R}}}^3} d^3{\textbf{k}}\,\,\,[2\omega ({\textbf{k}})]^{-1/2} \left\{ a^{\dagger }({\textbf{k}}) \left[ \displaystyle {\prod _{j=1}^{3}} \overline{\xi _{n^j}(k_j)} \right] e^{i\omega t} \right\} \\ \\ \phi ({\textbf{n}},t)= \phi ^{(-)}({\textbf{n}},t)+ \phi ^{(+)}({\textbf{n}},t) = \phi ^{\dagger }({\textbf{n}},t)\\ \\ \omega \equiv \omega ({\textbf{k}}) = k^{4}=-k_{4}=+\sqrt{{\textbf{k}}\cdot {\textbf{k}}+\mu ^2} > 0 \end{array} \end{aligned}$$

where these improper integrals are supposed to converge uniformly^24,25^. The linear operator $\phi ({\textbf{n}},t)$ representing Bosonic quanta are assumed to possess the relativistic four-momentum $(k^1,k^2,k^3,k^4)=(k_1,k_2,k_3,+\omega )=({\textbf{k}},+\omega ).$

The canonical quantization rules for operators that act on an infinite dimensional Hilbert space $a_{\mu }({\textbf{k}})$ and $a^{\dagger }_{\mu }({\textbf{k}})$ are assumed to be the commutators:

6 $$\begin{aligned} \begin{array}{l} [a({\textbf{k}}), a^{\dagger }(\hat{{\textbf{k}}})] = -[a^{\dagger }(\hat{{\textbf{k}}}), a({\textbf{k}})]= \delta ^3 ({\textbf{k}}-\hat{{\textbf{k}}}) {\textbf{I}}({\textbf{k}}) \\ \\ \; [a({\textbf{k}}), a(\hat{{\textbf{k}}})] = [a^{\dagger }({\textbf{k}}), a^{\dagger }((\hat{{\textbf{k}}})]={\textbf{0}}({\textbf{k}}) \end{array} \end{aligned}$$

where ${\textbf{I}}({\textbf{k}})$ and ${\textbf{0}}({\textbf{k}})$ are the identity and zero operators respectively and $\delta ^3 ({\textbf{k}}-\hat{{\textbf{k}}})$ is the Dirac distribution function^5,6^. These commutation relations imply the following commutators for our second quantized Bosonic field

7 $$\begin{aligned} \begin{array}{l} [\phi ^{(+)}({\textbf{n}},t), \phi ^{(+)}(\hat{{\textbf{n}}},{\hat{t}})] = [\phi ^{(-)}({\textbf{n}},t), \phi ^{(-)}(\hat{{\textbf{n}}},{\hat{t}})] ={\textbf{0}}\\ \\ \;[\phi ^{(-)}({\textbf{n}},t), \phi ^{(+)}(\hat{{\textbf{n}}},{\hat{t}})] = -i \Delta _{(+)}({\textbf{n}},t;\hat{{\textbf{n}}},{\hat{t}};\mu ) {\textbf{I}} \\ \\ \;[\phi ^{(+)}({\textbf{n}},t), \phi ^{(-)}(\hat{{\textbf{n}}},{\hat{t}})] = i \Delta _{(-)}({\textbf{n}},t;\hat{{\textbf{n}}},{\hat{t}};\mu ) {\textbf{I}} \\ \\ \;[\phi ({\textbf{n}},t), \phi ^{\dagger }(\hat{{\textbf{n}}},{\hat{t}})] = -i \Delta ({\textbf{n}},t;\hat{{\textbf{n}}},{\hat{t}};\mu ) {\textbf{I}} \\ \\ \end{array} \end{aligned}$$

Here, $\Delta _{(\pm )}({\textbf{n}},t;\hat{{\textbf{n}}},{\hat{t}};\mu )$ and $\Delta ({\textbf{n}},t;\hat{{\textbf{n}}},{\hat{t}};\mu )$ are non-singular Green’s functions for Eq. (4) as discussed in the Supplemental material.

## Second quantization of the free relativistic Fermionic anti-Fermionic partial difference-differential wave equation

Consider the following irreducible representation of $4 \times 4$ Dirac matrices with real and complex entries^13,24^

8 $$\begin{aligned} \begin{array}{l} \gamma ^{1}_{(4 \times 4)}:= \begin{bmatrix} 0 &{}\quad 0 &{}\quad 0 &{}\quad 1 \\ 0 &{}\quad 0 &{}\quad 1 &{}\quad 0 \\ 0 &{}\quad 1 &{}\quad 0 &{}\quad 0 \\ 1 &{}\quad 0 &{}\quad 0 &{}\quad 0 \\ \end{bmatrix}, \;\; \gamma ^{2}_{(4 \times 4)}:= \begin{bmatrix} 0 &{}\quad 0 &{}\quad 0 &{}\quad -i \\ 0 &{}\quad 0 &{}\quad i &{}\quad 0 \\ 0 &{}\quad -i &{}\quad 0 &{}\quad 0 \\ i &{}\quad 0 &{}\quad 0 &{}\quad 0 \\ \end{bmatrix} \\ \\ \gamma ^{3}_{(4 \times 4)}:= \begin{bmatrix} 0 &{}\quad 0 &{}\quad 1 &{}\quad 0 \\ 0 &{}\quad 0 &{}\quad 0 &{}\quad -1 \\ 1 &{}\quad 0 &{}\quad 0 &{}\quad 0 \\ 0 &{}\quad -1 &{}\quad 0 &{}\quad 0 \\ \end{bmatrix}, \;\; \gamma ^{4}_{(4 \times 4)}:= \begin{bmatrix} -i &{}\quad 0 &{}\quad 0 &{}\quad 0 \\ 0 &{}\quad -i &{}\quad 0 &{}\quad 0 \\ 0 &{}\quad 0 &{}\quad i &{}\quad 0 \\ 0 &{}\quad 0 &{}\quad 0 &{}\quad i \\ \end{bmatrix} \end{array} \end{aligned}$$

and their important properties

9 $$\begin{aligned} \begin{array}{l} \gamma ^{a\dagger } = \gamma ^{a}, \; \gamma ^{4\dagger } = -\gamma ^{4}\\ \\ \gamma ^{\mu } \gamma ^{\nu } + \gamma ^{\nu } \gamma ^{\mu } = 2 \eta ^{\mu \nu } I_{(4 \times 4)} \end{array} \end{aligned}$$

The Dirac bispinor wave field $\psi ({\textbf{n}},t)=[\psi ({\textbf{n}},t)]_{(4 \times 1)}$ is a $4 \times 1$ column vector field with entries of complex numbers. It physically represents a relativistic Fermion-anti-Fermion wave field defined over discrete phase space and continuous time. We introduce a corresponding conjugate $1 \times 4$ row wave field as follows,

10 $$\begin{aligned} \tilde{\psi }({\textbf{n}},t):= i \psi ^{\dagger }({\textbf{n}},t) \gamma ^{4} \end{aligned}$$

The discrete phase space continuous time Fermionic-anti-Fermionic wave equations are furnished by^13,24^

11 $$\begin{aligned} \begin{array}{l} \gamma ^a \Delta ^{\#}_{a} \psi ({\textbf{n}},t) + \gamma ^{4} \partial _t \psi ({\textbf{n}},t) + m\psi ({\textbf{n}},t) = 0_{(4 \times 1)} \\ \\ \; [\Delta ^{\#}_{a} \tilde{\psi }({\textbf{n}},t)]\gamma ^{a} + [\partial _t \tilde{\psi }({\textbf{n}},t)] \gamma ^{4} -m\tilde{\psi }({\textbf{n}},t) = 0_{(1 \times 4)} \end{array} \end{aligned}$$

Here, the positive constant $m>0$ represents the mass of the Fermionic-anti-Fermionic particle. These equations are essentially the partial difference-differential versions of the Dirac equation. We now explore a class of exact plane wave solutions by the following trial solution

12 $$\begin{aligned} \psi ({\textbf{n}},t) = \zeta ({\textbf{p}}, p_4) \left[ \prod _{j=1}^{3} \xi _{n^j}(p_j) \right] e^{ip_4 t} \end{aligned}$$

where $\zeta ({\textbf{p}}, p_4)$ is a $(4 \times 1)$-column vector function of four-momentum variables $({\textbf{p}}, p_4)$. The form of this trial wavefunction is chosen as it is relativistically invariant and leads to an exact solution as shown below. By substituting this trial wavefunction into the partial difference-differential Dirac equation above, we arrive at the following algebraic equations

13 $$\begin{aligned} \begin{array}{l} \eta ^{\mu \nu }p_{\mu }p_{\nu } +m^2 = 0,\\ \\ p^4=-p_4=\pm \sqrt{\delta ^{ab} p_a p_b +m^2} = \pm \sqrt{||{\textbf{p}}||^2 +m^2}\\ \\ E \equiv E({\textbf{p}}):= +\sqrt{{\textbf{p}} \cdot {\textbf{p}} +m^2} = +\sqrt{||{\textbf{p}}||^2 +m^2} >0 \end{array} \end{aligned}$$

and the following four linearly independent solutions

14 $$\begin{aligned} \begin{array}{l} \zeta _{(r)}({\textbf{p}}, p^4)=\zeta _{(r)}({\textbf{p}}, E)=: u_{(r)}({\textbf{p}}), \;\; E = E({\textbf{p}}) > 0 \\ \\ \zeta _{(r)}({\textbf{p}}, p_4)=\zeta _{(r)}({\textbf{p}}, -E)=: v_{(r)}({\textbf{p}}), \;\; -E = -E({\textbf{p}}) < 0 \\ \\ r \in \{1,2\} \end{array} \end{aligned}$$

where $r \in \{1,2\}$ physically represents the spin-up and down cases for the Fermionic-anti-Fermionic quantas. The four explicit $(4 \times 1)$ column vector solutions are listed below^13,24^

15 $$\begin{aligned} \begin{array}{l} u_{(1)}({\textbf{p}}) = [(m+E)/2E]^{1/2} \begin{bmatrix} 1 \\ 0 \\ -i(m+E)^{-1}p_3 \\ -i(m+E)^{-1}(p_1 +ip_2) \\ \end{bmatrix},\\ \\ u_{(2)}({\textbf{p}}) = [(m+E)/2E]^{1/2} \begin{bmatrix} 0 \\ 1 \\ -i(m+E)^{-1}(p_1 -ip_2) \\ i(m+E)^{-1}p_3 \\ \end{bmatrix},\\ \\ v_{(1)}({\textbf{p}}) = [(m+E)/2E]^{1/2} \begin{bmatrix} i(m+E)^{-1}p_3 \\ -i(m+E)^{-1}(p_1 +ip_2) \\ 1 \\ 0 \\ \end{bmatrix},\\ \\ v_{(2)}({\textbf{p}}) = [(m+E)/2E]^{1/2} \begin{bmatrix} i(m+E)^{-1}(p_1 -ip_2) \\ -i(m+E)^{-1}p_3 \\ 0 \\ 1 \\ \end{bmatrix}. \end{array} \end{aligned}$$

Here, $u_{(1)}({\textbf{p}})$ and $u_{(2)}({\textbf{p}})$ represent Fermionic quanta and $v_{(1)}({\textbf{p}})$ and $v_{(2)}({\textbf{p}})$ represent anti-Fermionic quanta solutions. The above solutions also satisfy the orthonormality conditions

16 $$\begin{aligned} \begin{array}{l} {\tilde{u}}_{(r)}({\textbf{p}}) \cdot u_{(s)}({\textbf{p}}) = - {\tilde{v}}_{(r)}({\textbf{p}}) \cdot v_{(s)}({\textbf{p}}) =\delta _{(rs)} \\ \\ {\tilde{u}}_{(r)}({\textbf{p}}) \cdot v_{(s)}({\textbf{p}}) = {\tilde{v}}_{(r)}({\textbf{p}}) \cdot u_{(s)}({\textbf{p}}) = 0 \end{array} \end{aligned}$$

In later sections, we will have to consider the case of very low values of three-momentum ${\textbf{p}}$. As explained in^26^, this is a justifiable approximation for external potential such as the Coulomb or Yukawa style potentials. Therefore, it useful to derive approximations of low external momenta $||{\textbf{p}}||$ to the above solutions. The following expansions to order $O \left( ||{\textbf{p}}||^4 \right)$ are

17 $$\begin{aligned} \begin{array}{l} E({\textbf{p}}) = m +\left( ||{\textbf{p}}||^2/2m \right) + O \left( ||{\textbf{p}}||^4 \right) , \\ \\ \; [m+E({\textbf{p}})/2E({\textbf{p}})]^{1/2} = [1-(1/2)\left( ||{\textbf{p}}||/2m \right) ^2 ]+O \left( ||{\textbf{p}}||^4 \right) \\ \\ u_{(1)} = \begin{bmatrix} 1\\ 0\\ 0\\ 0 \end{bmatrix} + \begin{bmatrix} -(1/2)\left( ||{\textbf{p}}||^2/2m \right) \\ 0 \\ -i(p_3/2m)[1-(3/2)\left( ||{\textbf{p}}||/2m \right) ^2 ] \\ -i\left( \dfrac{p_1+ip_2}{2m}\right) [1-(3/2)\left( ||{\textbf{p}}||/2m \right) ^2 ] \\ \end{bmatrix} + O \left( ||{\textbf{p}}||^4 \right) \\ \\ u_{(2)} = \begin{bmatrix} 0\\ 1\\ 0\\ 0 \end{bmatrix} + \begin{bmatrix} 0 \\ (1/2)\left( ||{\textbf{p}}||^2/2m \right) \\ -i\left( \dfrac{p_1-ip_2}{2m}\right) [1-(3/2)\left( ||{\textbf{p}}||/2m \right) ^2 ] \\ + i(p_3/2m)[1-(3/2)\left( ||{\textbf{p}}||/2m \right) ^2 ] \\ \end{bmatrix} + O \left( ||{\textbf{p}}||^4 \right) \end{array} \end{aligned}$$

A class of exact plane wave solutions of the Dirac partial difference-differential equations above is furnished by the following Fourier–Hermite integrals^7–9^,

18 $$\begin{aligned} \begin{array}{l} \psi _{(4 \times 1)}^{(-)}({\textbf{n}},t) = \displaystyle \int \limits _{{{\mathbb {R}}}^3} d^3{\textbf{p}}\,\,\,[m/E({\textbf{p}})]^{1/2} \left\{ \sum _{r=1}^{2}\alpha _{(r)}({\textbf{p}})u_{(r)}({\textbf{p}}) \left( \prod _{j=1}^{3} \xi _{n^j}(p_j) \right) e^{-iE t} \right\} \\ \\ \psi _{(4 \times 1)}^{(+)}({\textbf{n}},t) = \displaystyle \int \limits _{{{\mathbb {R}}}^3} d^3{\textbf{p}}\,\,\,[m/E({\textbf{p}})]^{1/2} \left\{ \sum _{r=1}^{2}\beta ^{\dagger }_{(r)}({\textbf{p}})v_{(r)}({\textbf{p}}) \left( \prod _{j=1}^{3} \overline{\xi _{n^j}(p_j)} \right) e^{iE t} \right\} \\ \\ \psi _{(4 \times 1)}({\textbf{n}},t) = \psi _{(4 \times 1)}^{(-)}({\textbf{n}},t) + \psi _{(4 \times 1)}^{(+)}({\textbf{n}},t) \\ \\ \tilde{\psi }_{(1 \times 4)}^{(-)}({\textbf{n}},t) = \displaystyle \int \limits _{{{\mathbb {R}}}^3} d^3{\textbf{p}}\,\,\,[m/E({\textbf{p}})]^{1/2} \left\{ \displaystyle \sum _{r=1}^{2}\alpha ^{\dagger }_{(r)}({\textbf{p}}){\tilde{u}}_{(r)}({\textbf{p}}) \left( \prod _{j=1}^{3} \overline{\xi _{n^j}(p_j)} \right) e^{iE t} \right\} \\ \\ \tilde{\psi }_{(1 \times 4)}^{(+)}({\textbf{n}},t) = \displaystyle \int \limits _{{{\mathbb {R}}}^3} d^3{\textbf{p}}\,\,\,[m/E({\textbf{p}})]^{1/2} \left\{ \displaystyle \sum _{r=1}^{2}\beta _{(r)}({\textbf{p}}){\tilde{v}}_{(r)}({\textbf{p}}) \left( \prod _{j=1}^{3} \xi _{n^j}(p_j) \right) e^{-iE t} \right\} \\ \\ \tilde{\psi }_{(1 \times 4)}({\textbf{n}},t) = \tilde{\psi }_{(1 \times 4)}^{(-)}({\textbf{n}},t) + \tilde{\psi }_{(1 \times 4)}^{(+)}({\textbf{n}},t) \end{array} \end{aligned}$$

The 4-component column vector $\psi _{(4 \times 1)}^{(-)}({\textbf{n}},t)$ and the 4-component row vector $\tilde{\psi }_{(1 \times 4)}^{(-)}({\textbf{n}},t)$ are associated with the Fermionic wave field whereas the vectors $\psi _{(4 \times 1)}^{(+)}({\textbf{n}},t)$ and $\tilde{\psi }_{(1 \times 4)}^{(+)}({\textbf{n}},t)$ are the anti-Fermionic wave fields.

Now, we shall introduce the canonical or second quantization of the free Fermionic-anti-Fermionic wave fields. The Fourier–Hermite coefficients $\alpha _{(r)}({\textbf{p}}), \alpha ^{\dagger }_{(r)}({\textbf{p}}), \beta _{(r)}({\textbf{p}}),\beta ^{\dagger }_{(s)}(\hat{{\textbf{p}}})$ act as linear operators that satisfy anti-commutation rules^10^,

19 $$\begin{aligned} \begin{array}{l} \; [A,B]_+ \equiv AB+BA=[B,A]_+ \\ \\ \; [\alpha _{(r)}({\textbf{p}}), \alpha _{(s)}(\hat{{\textbf{p}}})]_+ = [\beta _{(r)}({\textbf{p}}), \beta _{(s)}(\hat{{\textbf{p}}})]_+={\textbf{0}} \\ \\ \; [\alpha ^{\dagger }_{(r)}({\textbf{p}}), \alpha ^{\dagger }_{(s)}(\hat{{\textbf{p}}})]_+ = [\beta ^{\dagger }_{(r)}({\textbf{p}}), \beta ^{\dagger }_{(s)}(\hat{{\textbf{p}}})]_+= {\textbf{0}}\\ \\ \; [\alpha _{(r)}({\textbf{p}}), \beta _{(s)}(\hat{{\textbf{p}}})]_+ = [\alpha ^{\dagger }_{(r)}({\textbf{p}}), \beta ^{\dagger }_{(s)}(\hat{{\textbf{p}}})]_+= {\textbf{0}}\\ \\ \; [\alpha _{(r)}({\textbf{p}}), \beta ^{\dagger }_{(s)}(\hat{{\textbf{p}}})]_+ = [\alpha ^{\dagger }_{(r)}({\textbf{p}}), \beta _{(s)}(\hat{{\textbf{p}}})]_+= {\textbf{0}}\\ \\ \; [\alpha _{(r)}({\textbf{p}}), \alpha ^{\dagger }_{(s)}(\hat{{\textbf{p}}})]_+ = [\beta _{(r)}({\textbf{p}}), \beta ^{\dagger }_{(s)}(\hat{{\textbf{p}}})]_+ = \delta _{(rs)} \delta ^3 ({\textbf{p}}-\hat{{\textbf{p}}}) {\textbf{I}} \end{array} \end{aligned}$$

The key physical motivation for choosing operators $\alpha _{(r)}({\textbf{p}}),\alpha ^{\dagger }_{(s)}(\hat{{\textbf{p}}}), \beta _{(r)}({\textbf{p}}), \beta ^{\dagger }_{(s)}(\hat{{\textbf{p}}})$ satisfying anti-commutation relations in contrast to operators $a_{\mu }({\textbf{k}}), a^{\dagger }_{\nu }(\hat{{\textbf{k}}})$ satisfying commutation relations are due to the fact that Bosonic fields obey Bose–Einstein statistics, whereas the Fermionic fields obey Fermi–Dirac statistics similar to the continuous space-time quantum field theoretic formulation of Bosons and Fermions. The corresponding anti-commutation relations between our various quantized Fermionic and anti-Fermionic wave fields are given by

20 $$\begin{aligned} \begin{array}{l} \;[\psi ^{(-)}({\textbf{n}},t), {\psi }^{(+)}(\hat{{\textbf{n}}},{\hat{t}})]_+ = [\psi ^{(-)}({\textbf{n}},t), \tilde{\psi }^{(+)}(\hat{{\textbf{n}}},{\hat{t}})]_+ = [0]_{(4 \times 4)} \\ \\ \;[\tilde{\psi }^{(+)}({\textbf{n}},t), {\psi }^{(-)}(\hat{{\textbf{n}}},{\hat{t}})]_+ = [\tilde{\psi }^{(+)}({\textbf{n}},t), \tilde{\psi }^{(-)}(\hat{{\textbf{n}}},{\hat{t}})]_+ = [0]_{(4 \times 4)} \\ \\ \\ \;[\psi ^{(-)}({\textbf{n}},t), \tilde{\psi }^{(-)}(\hat{{\textbf{n}}},{\hat{t}})]_+ = i [S_{(+)}^{\#}({\textbf{n}}, t;\hat{{\textbf{n}}},{\hat{t}};m)]_{(4 \times 4)} \\ \\ \;[\tilde{\psi }^{(+)}({\textbf{n}},t), \psi ^{(+)}(\hat{{\textbf{n}}},{\hat{t}})]_+ = i [S_{(-)}^{\#}({\textbf{n}}, t;\hat{{\textbf{n}}},{\hat{t}};m)]_{(4 \times 4)} \\ \\ \end{array} \end{aligned}$$

The various non-singular Green’s functions $S_{(a)}^{\#}({\textbf{n}}, t ;\hat{{\textbf{n}}},{\hat{t}};m)$ will be elaborated further in the Supplemental material.

Let us prove the first non-trivial anti-commutation relation above starting with

21 $$\begin{aligned} \begin{array}{l} \;[\psi ^{(-)}({\textbf{n}},t), \tilde{\psi }^{(-)}(\hat{{\textbf{n}}},{\hat{t}})]_+ = \displaystyle \sum _{r=1}^{2}\displaystyle \sum _{s=1}^{2} \displaystyle \int \limits _{{{\mathbb {R}}}^3} \displaystyle \int \limits _{{{\mathbb {R}}}^3} d^3{\textbf{p}}d^3\hat{{\textbf{p}}}\,\,\,\left[ \dfrac{m}{\sqrt{E({\textbf{p}}){\hat{E}}(\hat{{\textbf{p}}})}}\right] \\ \\ \;[\alpha _{(r)}({\textbf{p}})\alpha ^{\dagger }_{(s)}(\hat{{\textbf{p}}})]_+[u_{(r)}({\textbf{p}}) {\tilde{u}}_{(s)}(\hat{{\textbf{p}}})] \displaystyle \left( \prod _{j=1}^{3} \xi _{n^j}(p_j) \overline{\xi _{{\hat{n}}^j}({\hat{p}}_j)} \right) e^{-iEt+i{\hat{E}}{\hat{t}}} \\ \\ or \\ \\ \;[\psi ^{(-)}({\textbf{n}},t), \tilde{\psi }^{(-)}(\hat{{\textbf{n}}},{\hat{t}})]_+ = \displaystyle \sum _{r=1}^{2} \displaystyle \int \limits _{{\mathbb R}^3}\left[ \dfrac{m}{E({\textbf{p}})}\right] [u_{(r)}({\textbf{p}}){\tilde{u}}_{(r)}({\textbf{p}})] \\ \displaystyle \prod _{j=1}^{3} \xi _{n^j}(p_j) \overline{\xi _{{\hat{n}}^j}(p_j)}e^{-iE(t-{\hat{t}})} d^3{\textbf{p}} \end{array} \end{aligned}$$

Now, it can be shown that^10^

22 $$\begin{aligned} \left[ \dfrac{m}{E({\textbf{p}})}\right] \displaystyle \sum _{r=1}^{2}[u_{(r)}({\textbf{p}}){\tilde{u}}_{(r)}({\textbf{p}})] = \left[ \dfrac{-i\gamma ^j p_j+i\gamma ^4E+mI}{2E} \right] _{(4 \times 4)} \end{aligned}$$

and therefore

23 $$\begin{aligned} \begin{array}{l} \;[\psi ^{(-)}({\textbf{n}},t), \tilde{\psi }^{(-)}(\hat{{\textbf{n}}},{\hat{t}})]_+ = \\ \ \displaystyle -\int \limits _{{{\mathbb {R}}}^3} \left[ \dfrac{i\gamma ^j p_j-i\gamma ^4E-mI}{2E} \right] \displaystyle \prod _{j=1}^{3} \xi _{n^j}(p_j) \overline{\xi _{{\hat{n}}^j}(p_j)}e^{-iE(t-{\hat{t}})} d^3{\textbf{p}} \end{array} \end{aligned}$$

where in the Supplemental material, it is shown the the Fermionic Green’s function is

24 $$\begin{aligned} \begin{array}{l} [S_{(+)}({\textbf{n}}, t;\hat{{\textbf{n}}},{\hat{t}};m)]_{(4 \times 4)} = \\ \\ i \displaystyle \int \limits _{{{\mathbb {R}}}^3} \left[ \dfrac{i\gamma ^j p_j-i\gamma ^4E-mI}{2E} \right] \displaystyle \prod _{j=1}^{3} \xi _{n^j}(p_j) \overline{\xi _{{\hat{n}}^j}(p_j)}e^{-iE(t-{\hat{t}})} d^3{\textbf{p}} \end{array} \end{aligned}$$

Therefore, the third relation of Eq. (20) follows. The other anti-commutation relations follow in a similar manner.

## Interactions of relativistic fields, the $S^{\#}$-matrix in discrete phase space-continuous time, and Møller Scattering

The purpose of this section is to obtain an expression for the potential between two Fermions exchanging one Boson resulting in a Yukawa style potential. This is derived by calculating the discrete phase space-continuous time $S^{\#}$-matrix. Discrete phase space-continuous time Feynman graph perturbation rules in four momentum space are presented and then used specifically to calculate the terms of the $S^{\#}$-matrix for Møller scattering in the low momentum approximation.

The relativistic Lagrangian of the second quantized interacting Fermionic and Bosonic fields is assumed to given by^7–9,12^

25 $$\begin{aligned} L_{int} ({\textbf{n}}, t):= -igN [ \tilde{\psi } ({\textbf{n}},t) \psi ({\textbf{n}},t) \phi ({\textbf{n}},t)]. \end{aligned}$$

Here, *g* represents the strong coupling constant between the Fermionic-anti-Fermionic field and a Bosonic field. Furthermore, $N[ \cdots ]$ stands for normal ordering. The scattering matrix in the background of discrete phase space and continuous time, denoted by $S^{\#}$-matrix, is defined by the operator-valued infinite series^7–12^

26 $$\begin{aligned} \begin{array}{l} S^{\#} = I + \sum \limits _{j=1}^{\infty } \dfrac{(g)^j}{j!} \sum \limits _{{\textbf{n}}^{1}=(0)} \cdots \sum \limits _{{\textbf{n}}^{j}=(0)} \displaystyle \int \limits _{\mathbb {R}} dt^{1} \\ \\ \cdots \displaystyle \int \limits _{\mathbb {R}} dt^{j} T \left\{ N[\tilde{\psi } ({\textbf{n}}^{1},t_{1}) \psi ({\textbf{n}}^{1},t^{1}) \phi ({\textbf{n}}^{1},t^{1})] \right. \\ \\ \cdots \left. N[\tilde{\psi } ({\textbf{n}}^{j},t^{j}) \psi ({\textbf{n}}^{1},t^{j}) \phi ({\textbf{n}}^{j},t^{j})] \right\} =: I + \displaystyle \sum _{j=1}^{\infty }S^{\#}_{(j)} \end{array} \end{aligned}$$

Here, *T* denotes Wick’s time ordering operation. We distinguish the scattering matrix by the notation $S^{\#}$-matrix from the usual notation of S-matrix in continuous space-time because the physics in the discrete phase space and continuous time is different from the physics in the space-time continuum.

Now, let us consider a particular physical process characterized by the initial Hilbert space state vector $| i \rangle$ and the final Hilbert space state vector $|f \rangle$. The $S^{\#}$-matrix elements for such a process is provided by

27 $$\begin{aligned} \langle f | S^{\#}| i \rangle = \langle f | i \rangle + \sum \limits _{j=1}^{\infty }\langle f | S^{\#}_{(j)}| i \rangle \end{aligned}$$

Furthermore, we need to define the following vertex distribution functions

28 $$\begin{aligned} \begin{array}{l} \delta ^{\#}_{(3)}({\textbf{p}},{\textbf{q}},{\textbf{k}}):= \displaystyle \sum _{n^1=0}^{\infty }\sum _{n^2=0}^{\infty }\sum _{n^3=0}^{\infty } \left[ \prod _{j=1}^{3} \xi _{n^j}(p_j) \xi _{n^j}(q_j) \xi _{n^j}(k_j) \right] \\ \\ \delta ^{\#}_{(3)}({\textbf{p}},-{\textbf{q}},-{\textbf{k}}):= \displaystyle \sum _{n^1=0}^{\infty }\sum _{n^2=0}^{\infty }\sum _{n^3=0}^{\infty } \left[ \prod _{j=1}^{3} \xi _{n^j}(p_j) \overline{\xi _{n^j}(q_j)} \overline{\xi _{n^j}(k_j)} \right] \end{array} \end{aligned}$$

The function $\delta ^{\#}_{(3)}({\textbf{p}},-{\textbf{q}},-{\textbf{k}})$ above is different from the standard delta function factor $(2 \pi )^3 \delta ^{3}({\textbf{p}},-{\textbf{q}},-{\textbf{k}})$ found in the usual theory in space-time continuum. Also, the problem of reducing Eq. (28) into much simpler functions is an unsolved problem.

We provide Feynman graph rules to evaluate succinctly each term of the $S^{\#}$-matrix series in Table 1.

**Table 1:**
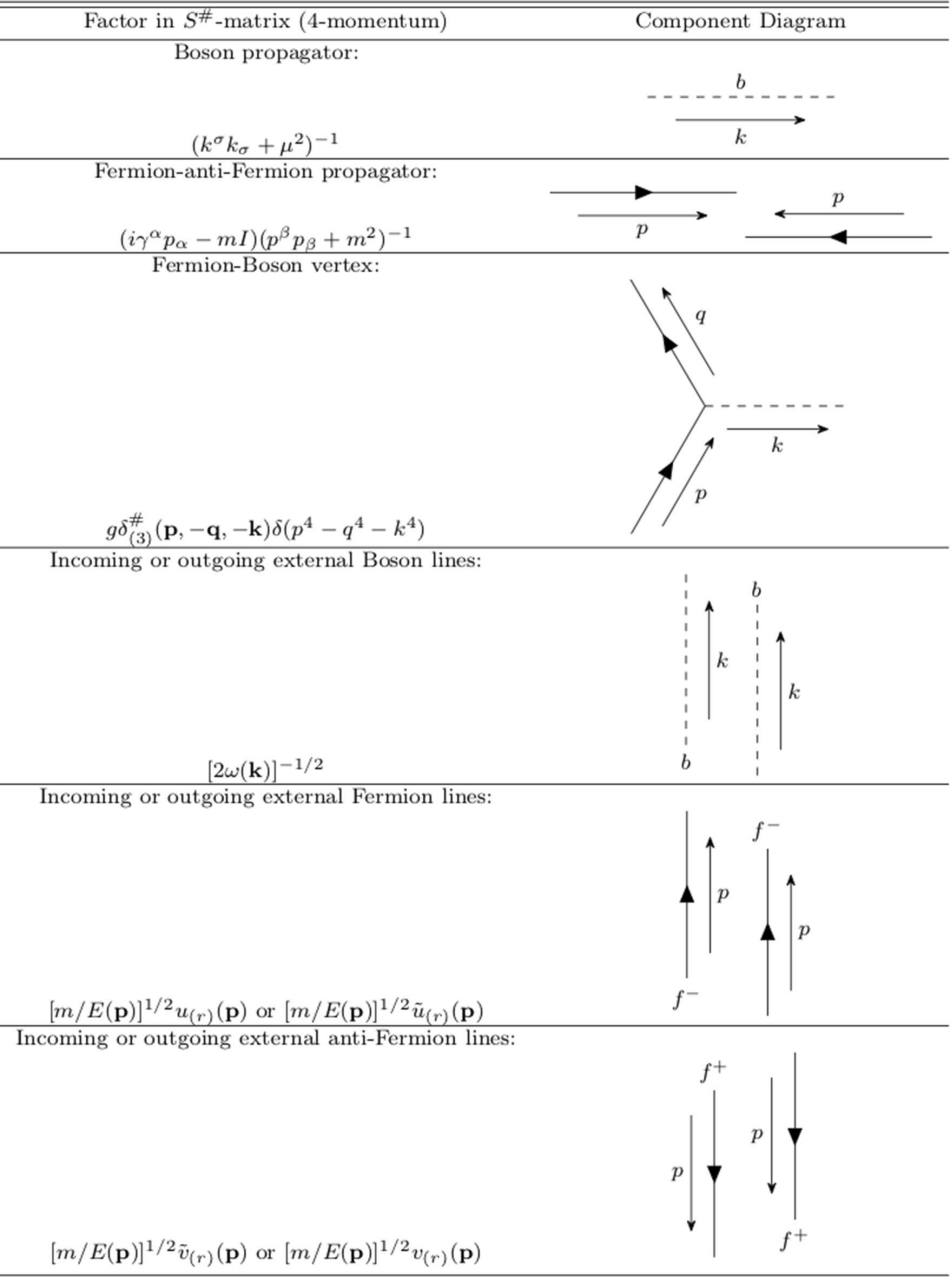
Feynman Graphs in four-momentum space.

Note that the relativistic Feynman prescriptions are identical between our $S^{\#}$-matrix and space-time continuum *S* matrix *except on the vertices*. At a vertex in our formalism, one has the relativistic term

29 $$\begin{aligned} g\delta ^{\#}_{(3)}({\textbf{p}},-{\textbf{q}},-{\textbf{k}})\delta (p^4-q^4-k^4). \end{aligned}$$

**Figure 1:**
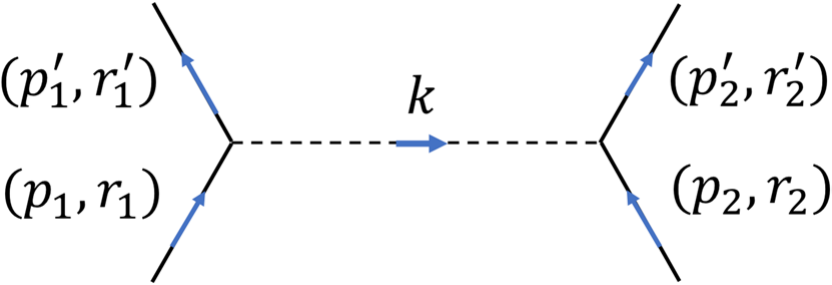
The Møller scattering of two Fermions by exchange of one Boson.

As an illustration of computing explicitly an element of$\langle f | S^{\#}_{(j)} - I | i \rangle$ in (27), using the corresponding component of the Feynman rules in Table 1, we consider the case of Møller scattering of two Fermions by exchange of one Boson as depicted in Fig. 1.

The element $\langle p^{'}_2 , p^{'}_1 | S^{\#}_{(2)} | p_1 , p_2 \rangle$ corresponding to Fig. 1 will be evaluated using the very low momentum approximation for the external Fermion lines as discussed above in (17). Following this, we obtain

30 $$\begin{aligned} \begin{array}{l} \langle p^{'}_2, p^{'}_1 | S^{\#}_{(2)} | p_1, p_2 \rangle \approx \left( \dfrac{g^2}{4\pi }\right) \left[ \dfrac{m^2}{\sqrt{E^{'}_1 E^{'}_2 E_1 E_2}} \right] \delta (E^{'}_2+E^{'}_1-E_2-E_1) \\ \\ \;[\delta _{(r'_1,r_1)}\delta _{(r'_2,r_2)}]\displaystyle \int \limits _{{\mathbb R}^3} \left\{ \delta ^{\#}_{(3)}(\mathbf {p_1},-\mathbf {p^{'}_1},-{\textbf{k}}) [{\textbf{k}} \cdot {\textbf{k}} +(\mu )^2]^{-1}\delta ^{\#}_{(3)}(\mathbf {p_2},-\mathbf {p^{'}_2},+{\textbf{k}})\right\} d^3{\textbf{k}} \\ \\ = \left( \dfrac{g^2}{4\pi }\right) \left[ \dfrac{m^2}{\sqrt{E^{'}_1 E^{'}_2 E_1 E_2}} \right] \delta (E^{'}_2+E^{'}_1-E_2-E_1)[\delta _{(r'_1,r_1)}\delta _{(r'_2,r_2)}] \\ \\ \displaystyle \int \limits _{{{\mathbb {R}}}^3}\biggl \{[{\textbf{k}} \cdot {\textbf{k}} +\mu ^2]^{-1} \left[ \displaystyle \sum _{{\textbf{n}}=0}^{\infty (3)} \prod _{a=1}^{3} \xi _{n^a}(p_{1a}) \overline{\xi _{n^a}(p'_{1a})} \overline{\xi _{n^a}(k_a)} \right] \\ \\ \left[ \displaystyle \sum _{\hat{{\textbf{n}}}=0}^{\infty (3)} \prod _{b=1}^{3} \xi _{{\hat{n}}^b}(p_{2b}) \overline{\xi _{{\hat{n}}^b}(p'_{2b})} \xi _{{\hat{n}}^b}(k_b)\right] \biggr \}d^3{\textbf{k}} \end{array} \end{aligned}$$

Inside the above equation, one may find the double Hermite transform for the Green’s function defined as

31 $$\begin{aligned} G^{\#}({\textbf{n}}, \hat{{\textbf{n}}};\mu ):= \displaystyle \int \limits _{{{\mathbb {R}}}^3}[{\textbf{k}} \cdot {\textbf{k}} +\mu ^2]^{-1} \left[ \displaystyle \prod _{j=1}^{3} \overline{\xi _{n^j}(k_j)}\xi _{{\hat{n}}^j}(k_j) \right] d^3{\textbf{k}} \end{aligned}$$

To compare and contrast the above result (31) with the usual second order S-matrix elements for Møller scattering over space-time continuum, we furnish from^10,13^

32 $$\begin{aligned} \begin{array}{l} \langle p^{'}_2, p^{'}_1 | S_{(2)} | p_1, p_2 \rangle \approx \left( \dfrac{g^2}{4\pi }\right) \left[ \dfrac{m^2}{\sqrt{E^{'}_1 E^{'}_2 E_1 E_2}} \right] \delta (E^{'}_2+E^{'}_1-E_2-E_1) \\ \\ \;[\delta _{(r'_1,r_1)}\delta _{(r'_2,r_2)}]\displaystyle \int \limits _{{\mathbb R}^3} \left\{ \delta ^3(\mathbf {p_1},-\mathbf {p^{'}_1},-{\textbf{k}}) [{\textbf{k}} \cdot {\textbf{k}} +(\mu )^2]^{-1}\delta ^3(\mathbf {p_2},-\mathbf {p^{'}_2},+{\textbf{k}})\right\} d^3{\textbf{k}} \\ \\ = \left( \dfrac{g^2}{4\pi }\right) \left[ \dfrac{m^2}{\sqrt{E^{'}_1 E^{'}_2 E_1 E_2}} \right] \delta (E^{'}_2+E^{'}_1-E_2-E_1)[\delta _{(r'_1,r_1)}\delta _{(r'_2,r_2)}] \\ \\ \displaystyle \int \limits _{{{\mathbb {R}}}^3}\biggl \{[{\textbf{k}} \cdot {\textbf{k}} +\mu ^2]^{-1} \left[ \dfrac{1}{(2\pi )^{3/2}}\displaystyle \int \limits _{{\mathbb R}^3}\exp \{-i(\mathbf {p_1}-\mathbf {p^{'}_1}-{\textbf{k}})\cdot \mathbf {x_1}\} d^3\mathbf {x_1}\right] \\ \\ \left[ \dfrac{1}{(2\pi )^{3/2}}\displaystyle \int \limits _{{\mathbb R}^3}\exp \{-i(\mathbf {p_2}-\mathbf {p^{'}_2}-{\textbf{k}})\cdot \mathbf {x_2}\} d^3\mathbf {x_2}\right] \biggr \}d^3{\textbf{k}} \end{array} \end{aligned}$$

The above equation may be regarded as the double Fourier transform for the Green’s function

33 $$\begin{aligned} G(\mathbf {x_1}, \mathbf {x_2};\mu ) = G(\mathbf {x_1}-\mathbf {x_2};\mu ):= \displaystyle \dfrac{1}{(2\pi )^{3}}\int \limits _{{\mathbb R}^3}[{\textbf{k}} \cdot {\textbf{k}} +\mu ^2]^{-1} \exp \{-i(\mathbf {x_1}-\mathbf {x_2})\cdot {\textbf{k}}\}d^3{\textbf{k}} \end{aligned}$$

with the limit

34 $$\begin{aligned} \lim _{\mathbf {x_1} \rightarrow \mathbf {x_2}} |G(\mathbf {x_1}-\mathbf {x_2};\mu ) | \rightarrow \infty \end{aligned}$$

This equation implies the following static partial differential equation

35 $$\begin{aligned} \begin{array}{l} \delta ^{ab} \partial _{(1)^a}\partial _{(1)^b}G(\mathbf {x_1}-\mathbf {x_2};\mu ) - \mu ^2 G(\mathbf {x_1}-\mathbf {x_2};\mu ) \\ \\ = \delta ^{ab} \partial _{(2)^a}\partial _{(2)^b}G(\mathbf {x_1}-\mathbf {x_2};\mu ) - \mu ^2 G(\mathbf {x_1}-\mathbf {x_2};\mu ) = -\delta ^{(3)}(\mathbf {x_1}-\mathbf {x_2}) \end{array} \end{aligned}$$

Now, the potential between two Fermions exchanging one Boson is furnished by,

36 $$\begin{aligned} V(\mathbf {x_1}-\mathbf {x_2}) = g^2 G(\mathbf {x_1}-\mathbf {x_2};\mu ^2) =\dfrac{g^2}{(2\pi )^2} \int \limits _{{{\mathbb {R}}}^3}[{\textbf{k}} \cdot {\textbf{k}} +\mu ^2]^{-1} \exp \{-i(\mathbf {x_1}-\mathbf {x_2})\cdot {\textbf{k}}\}d^3{\textbf{k}} \end{aligned}$$

To obtain a closed form solution, we transform to spherical polar coordinates

37 $$\begin{aligned} \begin{array}{l} {\textbf{x}}=\mathbf {x_1}-\mathbf {x_2}, \;\;\; r =|| {\textbf{x}} ||, \;\;\; k =|| {\textbf{k}} || \\ \\ {\textbf{x}} = (r\sin \theta \cos \phi , r \sin \theta \sin \phi , r \cos \theta ) \\ \\ {\textbf{k}} = (k\sin \hat{\theta }\cos \hat{\phi }, k \sin \hat{\theta }\sin \hat{\phi }, k \cos \hat{\theta }) \\ \\ \cos \gamma = \sin \theta \sin \hat{\theta }\cos \phi \cos \hat{\phi }+\sin \theta \sin \hat{\theta }\sin \phi \sin \hat{\phi }+\cos \theta \cos \hat{\theta }, \;\;\; 0 \le \gamma \le \pi \\ \\ {\textbf{x}} \cdot {\textbf{k}}=rk \cos \gamma =: rky \end{array} \end{aligned}$$

thereby obtaining

38 $$\begin{aligned} V(r) = \dfrac{g^2}{(2\pi )^2} \int \limits _0^\infty \int \limits _0^\pi \int \limits _{-\pi }^\pi [{\textbf{k}} \cdot {\textbf{k}} +\mu ^2]^{-1} e^{irk\cos \gamma }k^2 \sin \gamma dkd\gamma d\phi \end{aligned}$$

or

39 $$\begin{aligned} \begin{array}{l} V(r) = \dfrac{g^2}{(2\pi )^2} \displaystyle \int \limits _0^\infty \int \limits \limits _{-1}^1 [{\textbf{k}} \cdot {\textbf{k}} +\mu ^2]^{-1} e^{irky}k^2 dk dy \\ \\ =-i \dfrac{g^2}{(2\pi )^2} \left( \dfrac{1}{r} \right) \displaystyle \int \limits _0^\infty [{\textbf{k}} \cdot {\textbf{k}} +\mu ^2]^{-1} [e^{irk}-e^{-irk}]k dk \\ \\ = -i \dfrac{g^2}{(2\pi )^2} \left( \dfrac{1}{r} \right) \displaystyle \int \limits _0^\infty [{\textbf{k}} \cdot {\textbf{k}} +\mu ^2]^{-1} [2i\sin (rk)]k dk\\ \\ \end{array} \end{aligned}$$

Note that $[{\textbf{k}} \cdot {\textbf{k}} +\mu ^2]^{-1} [2i\sin (rk)]k$ is an even function whereas $[{\textbf{k}} \cdot {\textbf{k}} +\mu ^2]^{-1} [2i\cos (rk)]k$ is an odd function of *k*. Therefore we may write the above as

40 $$\begin{aligned} V(r)=-i \dfrac{g^2}{(2\pi )^2} \left( \dfrac{1}{r} \right) \displaystyle \int \limits _{-\infty }^\infty k[{\textbf{k}} \cdot {\textbf{k}} +\mu ^2]^{-1} e^{irk} dk \end{aligned}$$

The integrand has two simple poles at $k \pm i\mu$. Using a standard counter-clockwise contour *C* around the pole $k=i \mu$ in the upper half plane, the integral above using the Cauchy residue theorem is

41 $$\begin{aligned} \displaystyle \int \limits _{-\infty }^\infty k[{\textbf{k}} \cdot {\textbf{k}} +\mu ^2]^{-1} e^{irk} dk = \oint \limits _{C} k[{\textbf{k}} \cdot {\textbf{k}} +\mu ^2]^{-1} e^{irk} dk =-i\pi e^{-\mu r} \end{aligned}$$

Therefore, our potential is

42 $$\begin{aligned} V(r)=- \left( \dfrac{g^2}{4\pi } \right) \dfrac{e^{-\mu r}}{r} \end{aligned}$$

which is the well known *Yukawa potential* for the strong interaction between two protons exchanging a neutral meson. It is regular for $r >0$ but has a singularity at $r=0$. in Cartesian coordinates, the Yukawa potential for two interacting Fermions is

43 $$\begin{aligned} V(\mathbf {x_1},\mathbf {x_2};\mu )=- \left( \dfrac{g^2}{4\pi } \right) \dfrac{\exp (-\mu ||\mathbf 
{x_1}-\mathbf {x_2}||)}{||\mathbf {x_1}-\mathbf {x_2}||} \end{aligned}$$

In the case of one Fermion situated at the origin $\mathbf {x_2}=(0,0,0)$ and the other situated at $\mathbf {x_1}={\textbf{x}}$. the corresponding Yukawa potential is given by,

44 $$\begin{aligned} \begin{array}{l} V(\mathbf {x_1},\mathbf {x_2};\mu )=- \left( \dfrac{g^2}{4\pi } \right) \dfrac{\exp (-\mu ||{\textbf{x}}||)}{ ||{\textbf{x}}||} \\ \\ =- \left( \dfrac{g^2}{4\pi } \right) \dfrac{\exp (-\mu \sqrt{(x^1)^2+(x^3)^2+(x^3)^2})}{\sqrt{(x^1)^2+(x^3)^2+(x^3)^2}} \end{array} \end{aligned}$$

## Discrete phase space and a new non-singular Yukawa potential

In this section, we explicitly derive a non-singular Green’s function that is proportional to our new non-singular Yukawa potential as shown in the previous section. By using an incomplete gamma function, we show that this Green’s function is divergent free.

The static approximation to the scalar field Eq. (4) in the background of discrete phase space is furnished by the following partial difference equation

45 $$\begin{aligned} \delta ^{ab} \Delta _a^{\#}\Delta _b^{ \#}\phi ({\textbf{n}}) -\mu ^2 \phi ({\textbf{n}}) = 0 \end{aligned}$$

with a Green’s function given by

46 $$\begin{aligned} \begin{array}{l} G^{\#}({\textbf{n}}, \hat{{\textbf{n}}};\mu ) = G^{\#}(n^1,n^2,n^3,{\hat{n}}^1, {\hat{n}}^2,{\hat{n}}^3;\mu ) \\ \\ := \displaystyle \int \limits _{{{\mathbb {R}}}^3}[{\textbf{k}} \cdot {\textbf{k}} +(\mu )^2]^{-1} \left[ \displaystyle \prod _{j=1}^{3} \xi _{n^j}(k_j)\overline{\xi _{{\hat{n}}^j}(k_j)} \right] d^3{\textbf{k}} \end{array} \end{aligned}$$

By direct substitution of this Green’s function into the partial difference Eq. (45), one finds, using (2) and (3)

47 $$\begin{aligned} \begin{array}{l} \delta ^{ab} \Delta _a^{\#}\Delta _b^{ \#}G^{\#}({\textbf{n}}, \hat{{\textbf{n}}};\mu ) -\mu ^2 G^{\#}({\textbf{n}}, \hat{{\textbf{n}}};\mu ) \\ \\ = \delta ^{ab} \hat{\Delta }_a^{\#} \hat{\Delta }_b^{ \#}G^{\#}({\textbf{n}}, \hat{{\textbf{n}}};\mu ) -\mu ^2 G^{\#}({\textbf{n}}, \hat{{\textbf{n}}};\mu ) \\ \\ =-\delta _{(n^1 {{\hat{n}}}^1)}\delta _{(n^2 {{\hat{n}}}^2)}\delta _{(n^3 {{\hat{n}}}^3)} =: -\delta ^3_{({\textbf{n}} \hat{{\textbf{n}}})} \end{array} \end{aligned}$$

Furthermore, from using (2) and (3), one has from (46)

48 $$\begin{aligned} \begin{array}{l} G^{\#}({\textbf{n}}, {\textbf{0}};\mu ) = \left[ \dfrac{i^{n^1+n^2+n^3}}{\pi ^{3/2}2^{(n^1+n^2+n^3)/2}\sqrt{n^1!n^2!n^3!}} \right] \times \\ \displaystyle \int \limits _{{{\mathbb {R}}}^3}e^{-{\textbf{k}} \cdot {\textbf{k}}}[{\textbf{k}} \cdot {\textbf{k}} +\mu ^2]^{-1} \left[ \displaystyle \prod _{j=1}^{3} H_{n^j}(k_j) \right] d^3{\textbf{k}} \end{array} \end{aligned}$$

and

49 $$\begin{aligned} G^{\#}(n^1,0,0,0,0,0;\mu ) = \dfrac{i^{n^1}}{\pi ^{3/2}2^{\frac{n^1}{2}}\sqrt{n^1!}} \displaystyle \int \limits _{{{\mathbb {R}}}^3}e^{-{\textbf{k}} \cdot {\textbf{k}}}[{\textbf{k}} \cdot {\textbf{k}} +\mu ^2]^{-1} H_{n^1}(k_1) d^3{\textbf{k}} \end{aligned}$$

Now, we introduce spherical polar coordinates in three-momentum space

50 $$\begin{aligned} {\textbf{k}}=(k\cos \theta , k\sin \theta \cos \phi , k\sin \theta \sin \phi ), \;\;y=\cos \theta \end{aligned}$$

to transform (49) into

51 $$\begin{aligned} \begin{array}{l} G^{\#}(n^1,0,0,0,0,0;\mu ) = \dfrac{i^{n^1}}{\pi ^{3/2}2^{\frac{n^1}{2}}\sqrt{n^1!}} \times \\ \\ \displaystyle \int \limits _{0}^{\infty }\int \limits _{0}^{\pi }\int \limits _{-\pi }^{\pi } e^{-k^2} \;[k^2 +\mu ^2]^{-1} H_{n^1}(k\cos \theta )k^2 \sin \theta dk d\theta d\phi \\ \\ =\dfrac{i^{n^1}}{\sqrt{\pi } 2^{\frac{n^1}{2}-1}\sqrt{n^1!}}\displaystyle \int \limits _{0}^{\infty }\int \limits _{-1}^{1} e^{-k^2} \;[k^2 +\mu ^2]^{-1} H_{n^1}(ky)k^2 dkdy \end{array} \end{aligned}$$

Letting

52 $$\begin{aligned} x =k^2>0, \;\; k=+\sqrt{x} >0 \end{aligned}$$

(51) becomes

53 $$\begin{aligned} G^{\#}(n^1,0,0,0,0,0;\mu ) =\dfrac{i^{n^1}}{\sqrt{\pi } 2^{\frac{n^1}{2}}\sqrt{n^1!}}\displaystyle \int \limits _{0}^{\infty }\int \limits _{-1}^{1} e^{-x} \;[x +\mu ^2]^{-1} H_{n^1}(\sqrt{x}y)\sqrt{x}dxdy \end{aligned}$$

The coincidence limit of $n^1 \rightarrow 0$ of (53) yields^15^

54 $$\begin{aligned} G^{\#}(0,0,0,0,0,0;\mu ) = \mu \; e^{\mu ^2}\Gamma \left( -\dfrac{1}{2}, \mu ^2 \right) \end{aligned}$$

Here, the incomplete gamma function is defined by^15^

55 $$\begin{aligned} \begin{array}{l} \Gamma \left( -\dfrac{1}{2}, \mu ^2 \right) := \displaystyle \int \limits _{\mu ^2}^{\infty } w^{-3/2}e^{-w}dw \\ \\ \dfrac{\partial }{\partial \mu ^2}\Gamma \left( -\dfrac{1}{2}, \mu ^2 \right) =-\mu ^3 e^{-\mu ^2} \end{array} \end{aligned}$$

By the above three equations, it has been clearly demonstrated that the function $G^{\#}(0,0,0,0,0,0;\mu )$ is *divergence-free* giving us a new non-singular Yukawa potential as $V=g^2 G^{\#}$. This is described further in “section Various discrete phase space Green’s functions and a new non-singular Yukawa potential” below.

## Discrete phase space, a new non-singular Coulomb potential, and Beta functions

The Coulomb potential may be obtained by setting $\mu =0$ in the previous section. The Green’s function from (46) becomes

56 $$\begin{aligned} \begin{array}{l} G^{\#}({\textbf{n}}, \hat{{\textbf{n}}};0) = G^{\#}(n^1,n^2,n^3,{\hat{n}}^1, {\hat{n}}^2,{\hat{n}}^3;0) \\ \\ := \displaystyle \int \limits _{{{\mathbb {R}}}^3}[{\textbf{k}} \cdot {\textbf{k}}]^{-1} \left[ \displaystyle \prod _{j=1}^{3} \xi _{n^j}(k_j)\overline{\xi _{{\hat{n}}^j}(k_j)} \right] d^3{\textbf{k}} \end{array} \end{aligned}$$

or using Hermite polynomials

57 $$\begin{aligned} \begin{array}{l} G^{\#}({\textbf{n}}, {\textbf{0}};0) = \left[ \dfrac{i^{n^1+n^2+n^3}}{\pi ^{3/2}2^{(n^1+n^2+n^3)/2}\sqrt{n^1!n^2!n^3!}} \right] \times \\ \displaystyle \int \limits _{{{\mathbb {R}}}^3}e^{-{\textbf{k}} \cdot {\textbf{k}}}[{\textbf{k}} \cdot {\textbf{k}}]^{-1} \left[ \displaystyle \prod _{j=1}^{3} H_{n^j}(k_j) \right] d^3{\textbf{k}} \end{array} \end{aligned}$$

and

58 $$\begin{aligned} G^{\#}(n^1,0,0,0,0,0;0) = \dfrac{i^{n^1}}{\pi ^{3/2}2^{\frac{n^1}{2}}\sqrt{n^1!}} \displaystyle \int \limits _{{{\mathbb {R}}}^3}e^{-{\textbf{k}} \cdot {\textbf{k}}}[{\textbf{k}} \cdot {\textbf{k}}]^{-1} H_{n^1}(k_1) d^3{\textbf{k}} \end{aligned}$$

Once again, using the spherical coordinates of (50), we deduce that

59 $$\begin{aligned} G^{\#}(n^1,0,0,0,0,0;0) =\dfrac{i^{n^1}}{\sqrt{\pi } 2^{(\frac{n^1}{2}-1)}\sqrt{n^1!}}\displaystyle \int \limits _{0}^{\infty }\int \limits _{-1}^{1} e^{-k^2} H_{n^1}(ky) dkdy \end{aligned}$$

and

60 $$\begin{aligned} G^{\#}(2n^1,0,0,0,0,0;0) =\dfrac{(-1)^{n^1}}{\sqrt{\pi } 2^{(n^1-1)}\sqrt{(2n^1)!}}\displaystyle \int \limits _{0}^{\infty }\int \limits _{-1}^{1} e^{-k^2} H_{2n^1}(ky) dkdy \end{aligned}$$

and

61 $$\begin{aligned} \begin{array}{l} G^{\#}(2n^1+1,0,0,0,0,0;0) =\dfrac{i(-1)^{n^1}}{\sqrt{\pi } 2^{(n^1-1/2)}\sqrt{(2n^1+1)!}} \times \\ \displaystyle \int \limits _{0}^{\infty }\int \limits _{-1}^{1} e^{-k^2} H_{2n^1+1}(ky) dkdy \end{array} \end{aligned}$$

As $H_{2n^1}(ky)$ and $H_{2n^1+1}(ky)$ are even and odd functions respectively of the variable *ky*, it is clear that

62 $$\begin{aligned} \begin{array}{l} \displaystyle \int \limits _{0}^{\infty }\int \limits _{-1}^{1} e^{-k^2} H_{2n^1}(ky) dkdy = \dfrac{1}{2}\displaystyle \int \limits _{-\infty }^{\infty }\int \limits _{-1}^{1} e^{-k^2}H_{2n^1}(ky) dkdy\\ \\ and \\ \\ \displaystyle \int \limits _{0}^{\infty }\int \limits _{-1}^{1} e^{-k^2} H_{2n^1+1}(ky)(ky) dkdy =0 \end{array} \end{aligned}$$

Furthermore, from^15^, using the relation

63 $$\begin{aligned} \displaystyle \int \limits _{-\infty }^{\infty } e^{-k^2} H_{2n^1}(ky) dk = (-1)^{n^1}\sqrt{\pi }\dfrac{(2n^1)!}{(n^1!)}(1-y^2)^{n^1} \end{aligned}$$

our Green’s functions reduce to

64 $$\begin{aligned} \begin{array}{l} G^{\#}(2n^1,0,0,0,0,0;0) =\left[ \dfrac{\sqrt{(2n^1)!}}{2^{n^1}(n^1!)}\right] \displaystyle \int \limits _{-1}^{1}(1-y^2)^{n^1}dy \\ \\ and \\ \\ G^{\#}(2n^1+1,0,0,0,0,0;0) = 0 \end{array} \end{aligned}$$

The integral above is the Euler beta function^15^

65 $$\begin{aligned} \begin{array}{l} \displaystyle \int \limits _{-1}^{1}(1-y^2)^{n^1}dy = 2^{(2n^1+1)}B(n^1+1,n^1+1)\\ \\ =2^{(2n^1+1)} \dfrac{[\Gamma (n^1+1)]^2}{\Gamma (2n^1+2)} =2^{(2n^1+1)}\dfrac{[(n^1)!]^2}{[(2n^1+1)!]} \end{array} \end{aligned}$$

leaving us with

66 $$\begin{aligned} G^{\#}(2n^1,0,0,0,0,0;0) =\dfrac{2^{(n^1+1)}\;(n^1)! \;\sqrt{(2n^1)!}}{(2n^1+1)!}=\dfrac{2^{(n^1+1)}\;(n^1)!}{(2n^1+1)\sqrt{(2n^1)!}} \end{aligned}$$

as was initially derived in^13^. In the coincidence limit $n^1 \rightarrow 0_+$, we have

67 $$\begin{aligned} G^{\#}(0,0,0,0,0,0;0) = +2 \end{aligned}$$

indicating that the discrete phase space Coulomb potential has no singularity as was derived in a different manner in^13^.

## Various discrete phase space Green’s functions and a new non-singular Yukawa potential

Here, we summarize the various discrete phase space and continuous time Green’s functions and potentials in a special way. From (46), we have

68 $$\begin{aligned} G^{\#}({\textbf{n}}, \hat{{\textbf{n}}};\mu ) = \displaystyle \int \limits _{{{\mathbb {R}}}^3}[{\textbf{k}} \cdot {\textbf{k}} +(\mu )^2]^{-1} \left[ \displaystyle \prod _{j=1}^{3} \xi _{n^j}(k_j)\overline{\xi _{{\hat{n}}^j}(k_j)} \right] d^3{\textbf{k}} \end{aligned}$$

and it’s various special cases

69 $$\begin{aligned} \begin{array}{l} (i) \; G^{\#}({\textbf{n}}, {\textbf{0}};\mu ) = \left[ \dfrac{i^{n^1+n^2+n^3}}{\pi ^{3/2}2^{(n^1+n^2+n^3)/2}\sqrt{n^1!n^2!n^3!}} \right] \\\\ \times  \displaystyle \int \limits _{{{\mathbb {R}}}^3}e^{-{\textbf{k}} \cdot {\textbf{k}}}[{\textbf{k}} \cdot {\textbf{k}} +\mu ^2]^{-1} \left[ \displaystyle \prod _{j=1}^{3} H_{n^j}(k_j) \right] d^3{\textbf{k}} \\ \\ (ii) \; G^{\#}(n^1,0,0,0,0,0;\mu ) = \dfrac{i^{n^1}}{\pi ^{3/2}2^{\frac{n^1}{2}}\sqrt{n^1!}} \displaystyle \int \limits _{{{\mathbb {R}}}^3}e^{-{\textbf{k}} \cdot {\textbf{k}}}[{\textbf{k}} \cdot {\textbf{k}} +\mu ^2]^{-1} H_{n^1}(k_1) d^3{\textbf{k}} \\ \\ (iii) \; G^{\#}(2n^1,0,0,0,0,0;\mu ) = \dfrac{(-1)^{n^1}}{\pi ^{3/2}2^{n^1}\sqrt{(2n^1)!}} \displaystyle \int \limits _{{{\mathbb {R}}}^3}e^{-{\textbf{k}} \cdot {\textbf{k}}}[{\textbf{k}} \cdot {\textbf{k}} +\mu ^2]^{-1} H_{2n^1}(k_1) d^3{\textbf{k}} \\ \\ (iv) \; G^{\#}(2n^1+1,0,0,0,0,0;\mu )=0 \\ \\ (v) \; G^{\#}({\textbf{n}}, {\textbf{0}};0) = \left[ \dfrac{i^{n^1+n^2+n^3}}{\pi ^{3/2}2^{(n^1+n^2+n^3)/2}\sqrt{n^1!n^2!n^3!}} \right]\\\\ \times  \displaystyle \int \limits _{{{\mathbb {R}}}^3}e^{-{\textbf{k}} \cdot {\textbf{k}}}[{\textbf{k}} \cdot {\textbf{k}}]^{-1} \left[ \displaystyle \prod _{j=1}^{3} H_{n^j}(k_j) \right] d^3{\textbf{k}} \\ \\ (vi) \; G^{\#}(n^1,0,0,0,0,0;0) = \dfrac{i^{n^1}}{\pi ^{3/2}2^{\frac{n^1}{2}}\sqrt{n^1!}} \displaystyle \int \limits _{{{\mathbb {R}}}^3}e^{-{\textbf{k}} \cdot {\textbf{k}}}[{\textbf{k}} \cdot {\textbf{k}}]^{-1} H_{n^1}(k_1) d^3{\textbf{k}} \\ \\ (vii) \; G^{\#}(2n^1,0,0,0,0,0;0) = \dfrac{(-1)^{n^1}}{\pi ^{3/2}2^{n^1}\sqrt{(2n^1)!}} \displaystyle \int \limits _{{{\mathbb {R}}}^3}e^{-{\textbf{k}} \cdot {\textbf{k}}}[{\textbf{k}} \cdot {\textbf{k}}]^{-1} H_{2n^1}(k_1) d^3{\textbf{k}} \\ \\ (viii) \; G^{\#}(2n^1+1,0,0,0,0,0;0)=0 \end{array} \end{aligned}$$

To reduce the notation, let us define $W^{\#}(n,\mu ) := G^{\#}(n,0,0;0,0,0;\mu )$, i.e. $W^{\#}(2n^1,\mu )=G^{\#}(2n^1,0,0;0,0,0;\mu )$ and compare the discrete formalism to the space-time continuum function implied by Eq. (42)

70 $$\begin{aligned} W(x^1;\mu )=G(x^1,0,0;0,0,0; \mu ) = \dfrac{1}{4\pi } \; \dfrac{e^{-\mu |x^1|}}{|x^1|} \end{aligned}$$

and the Coulomb potential

71 $$\begin{aligned} W(x^1;0)=G(x^1,0,0;0,0,0;0) = \dfrac{1}{4\pi } \; \dfrac{1}{|x^1|} \end{aligned}$$

Note that the actual potential function *V* between two Fermions interacting via a Boson must include the coupling coefficient *g* and *e*, i.e.

72 $$\begin{aligned} V(x^1;\mu )= W(x^1;\mu )= \dfrac{e^2}{4\pi } \; \dfrac{e^{-\mu |x^1|}}{|x^1|} \end{aligned}$$

and

73 $$\begin{aligned} V^{\#}(n^1;\mu )=-g^2 W^{\#}(n^1,\mu )= \dfrac{-g^2 \;i^{n^1}}{\pi ^{3/2}2^{\frac{n^1}{2}}\sqrt{n^1!}} \displaystyle \int \limits _{{{\mathbb {R}}}^3}e^{-{\textbf{k}} \cdot {\textbf{k}}}[{\textbf{k}} \cdot {\textbf{k}} +\mu ^2]^{-1} H_{n^1}(k_1) d^3{\textbf{k}} \end{aligned}$$

**Figure 2:**
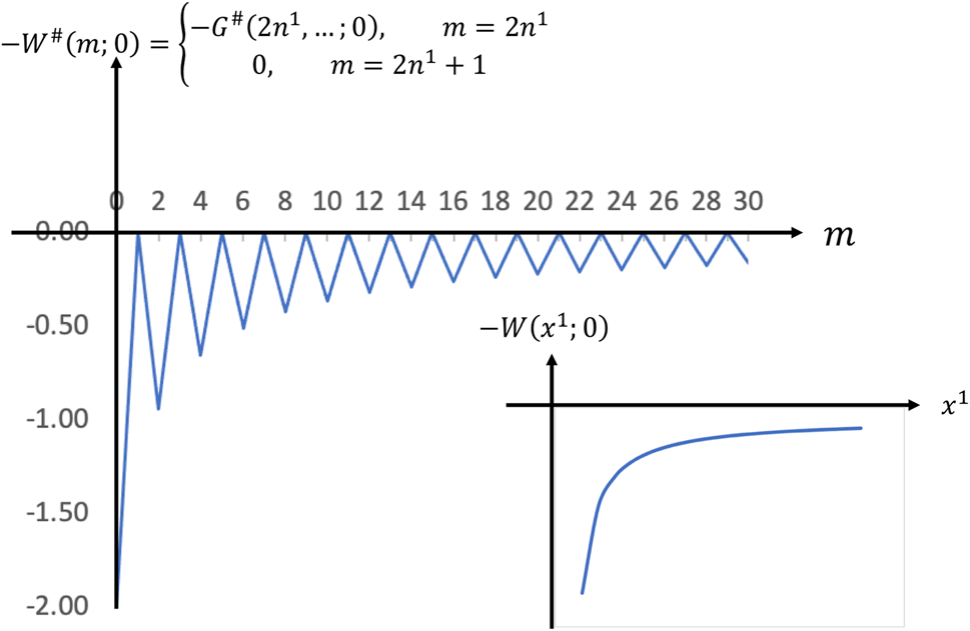
The negative of the usual singular Coulomb potential function $W(x^1;0)$ versus its non-singular counterpart $W^{\#}(m;0)$.

It is clear that continuum space-time function $W(x^1;\mu )= \frac{1}{4\pi } \; \frac{e^{-\mu |x^1|}}{|x^1|}$ from above has a singularity at $x^1=0$ whereas the discrete phase space version

74 $$\begin{aligned} \boxed { W^{\#}(2n^1;0) =\dfrac{2^{(n^1+1)}\;(n^1)!}{(2n^1+1)\sqrt{(2n^1)!}} } \end{aligned}$$

has no singularities even at $n^1 \rightarrow 0_+$ as shown in Fig. 2. We have already shown that $W^{\#}(n^1;\mu )$ is non-singular in Eqs. (54) and (55).

## Concluding remarks

In this paper, we have conducted an investigation into potential theory within discrete phase space and continuous time for a second quantized system of interacting relativistic Fermions and Bosons. The core mathematical formulation involves partial difference equations. Our analysis reveals that the Green’s functions and their associated potentials are free from singularities for the special case of proton-proton Møller scattering. In the approximation of very low external three-momenta, a new Yukawa potential is explicitly derived and rigorously proved to be divergence free.

Furthermore, the operators responsible for generating these Green’s functions offer an exact portrayal of quantum mechanics offering a new perspective in this field. This sets our formulation apart from conventional discrete theories, which typically emerge as approximations of continuous systems. It is worth noting that singular Green’s functions can lead to problematic divergences in S-matrix elements in traditional relativistic quantum field theory. However, with the relativistic version of the discrete representation we present in this work, the corresponding S-matrix elements remains finite leading directly to our new non-singular Yukawa potential.

### Supplementary Information

Supplementary Information.

## Data Availability

All data generated or analysed during this study are included in this published article [and its supplementary information files].
