# Supplementary figures and images for: Discrete phase space-continuous time relativistic Klein–Gordon and Dirac equations, and a new non-singular Yukawa potential
Source: Sci Rep. 2023 Nov 21;13:20356. doi: 10.1038/s41598-023-47344-w (PMC10663489; doi:10.1038/s41598-023-47344-w)

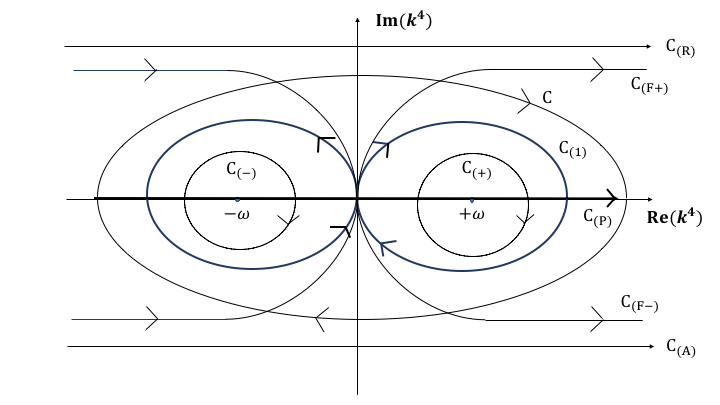

Supplement: Supplementary file 1 — Supplementary Information. [file 41598_2023_47344_MOESM1_ESM.zip › Supplemental/SupplementalFig1.png]

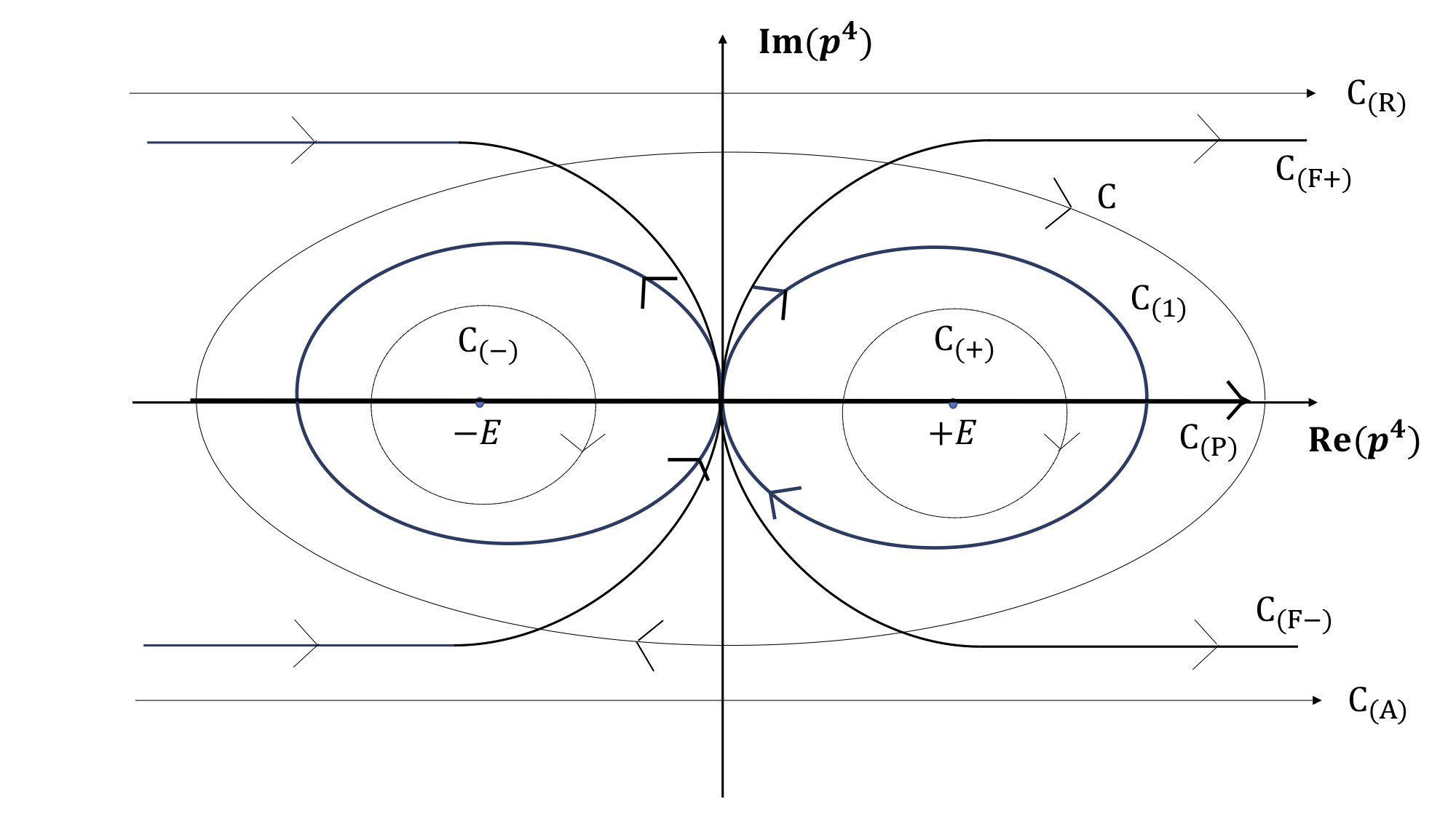

Supplement: Supplementary file 1 — Supplementary Information. [file 41598_2023_47344_MOESM1_ESM.zip › Supplemental/SupplementalFig2.png]
